# Source-Oriented Health Risk Assessment of Potentially Toxic Elements in the Water-Soil-Crop System Using Monte Carlo Simulation: A Case Study of the Laoguan River Basin, China

**DOI:** 10.3390/toxics13110952

**Published:** 2025-11-04

**Authors:** Xiaolin Jia, Hui Fu, Ding Ding, Xi Ren, Pei Zhao, Xidong Chen, Xiaonan Luo, Baojian Guo, Hongbin Xu, Zhiwei Sheng, Haitao Huang

**Affiliations:** 1 School of Human Settlements, North China University of Water Resources and Electric Power, Zhengzhou 450046, China; jiaxiaolin@ncwu.edu.cn (X.J.); zhaopei@ncwu.edu.cn (P.Z.); 2 Huanghuai Laboratory, Zhengzhou 450046, China; fuhui@hnas.ac.cn (H.F.); guobj@hnas.ac.cn (B.G.); 3 Henan Zhuoyue Construction Engineering Co., Ltd., Zhengzhou 450016, China; dean66778@163.com (D.D.); renxi1204@163.com (X.R.); weiweb@163.com (Z.S.); poormanxy@163.com (H.H.); 4 Faculty of Architecture, The University of Hong Kong, Hong Kong SAR 999007, China; xdchenrs@hku.hk; 5 School of Ecology and Environment, Zhengzhou University, Zhengzhou 450001, China; xuhongbin_gy@zzu.edu.cn

**Keywords:** mining-related contamination, multiple environmental media, probabilistic health risk, anthropogenic driving factor

## Abstract

Mining and smelting release potentially toxic elements (PTEs) that threaten ecosystems and public health. However, comprehensive risk assessments of PTEs across environmental media near mining areas remain scarce. The Laoguan River Basin is located in southwestern Henan Province, China. It lies within the water source area of China’s South-to-North Water Diversion Middle Route Project. This area has high geographic and ecological importance. In this study, we analyzed the pollution characteristics of PTEs in the water–soil–crop system. We also performed a source-oriented health risk assessment by integrating Monte Carlo simulation with source apportionment. According to this study, Mo and Sb were the predominant contaminants in soils and water. Pb, Cr, and Ni were elevated in crops. The health risk assessment indicated that PTEs in surface water were at acceptable levels. In contrast, PTEs in soils pose both non-carcinogenic and carcinogenic risks, particularly to children. The estimated risks were 1% (non-carcinogenic) and 64% (carcinogenic), with ingestion as the primary exposure pathway. Source apportionment showed that the surface water pollution was mainly linked to diverse mining activities. Soil pollution was jointly influenced by the geological background and mining and agricultural activities. Crop pollution was primarily associated with mining and agricultural activities. Geological background and mining were the main driving factors of the increased health risks for children. They accounted for 83% of the non-carcinogenic risk and 79% of the carcinogenic risk. Overall, these results are crucial for pollution control, safeguarding public health and safety, and promoting balanced economic and ecological development.

## 1. Introduction

Contamination by potentially toxic elements (PTEs) poses enduring risks to ecosystems and public health. As a result, it has received broad attention worldwide. Three-quarters of the elements in the periodic table are metals, but only a few are essential for living organisms [1]. Non-essential elements—such as cadmium (Cd), lead (Pb), antimony (Sb), and arsenic (As)—can harm environmental and human health even at relatively low concentrations [2,3,4]. In addition, some essential trace elements—such as copper (Cu), chromium (Cr), zinc (Zn), vanadium (V), nickel (Ni), and molybdenum (Mo)—become toxic in increased concentrations [5,6,7,8]. Various international organizations have identified PTEs as critical environmental pollutants [9,10]. This problem is particularly pronounced in areas with intensive mining and smelting activities [11,12,13]. In recent years, China has strengthened environmental governance. Many mining and smelting facilities have been closed, and remedial measures have been initiated to reduce the risk to nearby residents [14,15]. Nevertheless, PTE contamination from tailings continues to affect areas near abandoned mines and in remote locations. Rainfall, surface runoff, physical decomposition, and biogeochemical processes can all cause leaching of PTEs from tailings. These processes promote the migration of PTEs into surrounding water, soils, and ecosystems, consequently posing sustained health risks through food webs and other exposure pathways [16,17,18,19,20].

Currently, the assessment of residual PTE pollution and human health risks across different environmental media in mining areas is inadequate [21]. The long-term ecological impacts remain poorly understood. Clarifying the characteristics, ecological and health risks, and pollution sources of PTEs in such areas is essential for pollution control and safeguarding public safety [22,23,24]. Existing pollution assessment studies have primarily used the Nemerow index (NI), potential ecological risk index (PERI), and deterministic health risk assessment (HRA) model [25,26,27,28]. However, these deterministic methods often overlook regional environmental heterogeneity and uncertainties related to exposure parameters, potentially leading to biased results [29,30,31]. Probabilistic HRA using Monte Carlo simulation can effectively characterize these uncertainties, enhancing the practical significance of the assessment [32,33]. Furthermore, many investigations focus on a single medium (e.g., water or soil) and neglect the comprehensive effects of contaminants across multiple media [34,35,36]. To address these gaps, we integrated Monte Carlo simulation and source apportionment, and evaluated multiple environmental media, including surface water, soil, and crops.

The Laoguan River Basin is a critical water source for China’s South-to-North Water Diversion Middle Route Project, and thus it has important ecological significance. The upper reaches of the basin are mineral-rich, and long-term intensive mining and smelting have caused substantial PTE pollution. Thus, in this study, we focused on the surface water, agricultural soil, and major crops in this basin as the main research objects. Our objectives were (1) to analyze the pollution characteristics of various PTEs within the water-soil-crop system; (2) to assess the human health risks using a combination of Monte Carlo simulation and the HRA model, revealing the probability distribution of the health risks in the region; and (3) to identify the pollution sources using principal component analysis (PCA) and quantify the contributions of different sources to the health risks. The results provide valuable scientific support for PTE managing, regional environmental governance, and rational land-use planning in the similar mining areas.

## 2. Materials and Methods

### 2.1. Study Area

**Figure 1:**
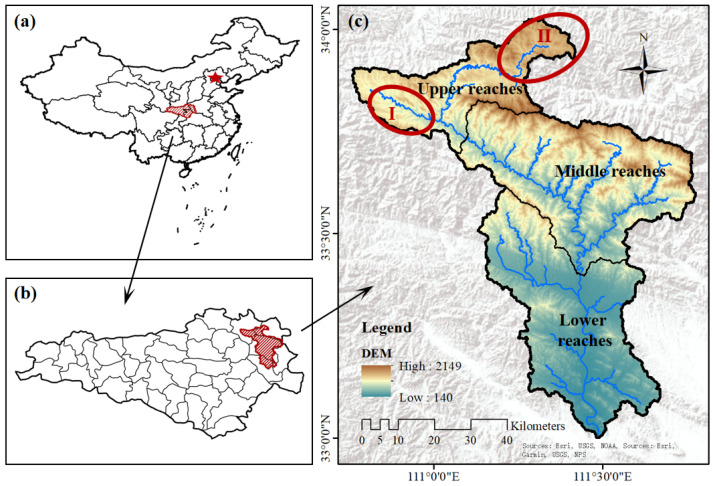
Location of the study area: (**a**) administrative divisions in China; (**b**) water source areas of China’s South-to-North Water Diversion Middle Route Project; (**c**) the study area. The red circles indicate the mining areas. The red five-pointed star indicates Beijing, China.

The Laoguan River Basin is located in the southwestern part of Henan Province, China, at the junction of Hubei, Shaanxi, and Henan provinces (110°17′30″–111°50′00″ E, 32°55′40″–34°01′01″ N) (Figure 1). The terrain in the basin slopes downward from north to south. Rivers and mountains interlace, and soil erosion is severe. Intensive mining and smelting activities in the upper reaches of the basin pose substantial threats to local water bodies, soils, and ecosystems. Mining Area (I) on the left is dominated by Sb. It has ceased extraction and is under environmental remediation. Mining Area (II) on the right is dominated by Pb and Zn. It remains in service, complies with national regulations, and is a major source of regional income. Several small Mo and V mines in the middle reaches have been decommissioned. Based on these mining characteristics, this study targeted the primary mined commodities (Sb, Mo, V, Pb, and Zn). We also examined their co-occurring elements—Cr, Ni, Cu, As, Cd, and Hg. Hg was detected only in soils (0.03–0.19 mg kg^−1^) and was below the detection limit in the other media. Subsequent pollution assessments therefore focus on the other ten PTEs. Agriculture is widespread in the basin, is small-scale and spatially scattered, and relies largely on surface-water irrigation. At the time of soil sampling, most crops were vegetables. To obtain a full understanding of the PTE status in the area, we evaluated the PTE contamination across multiple environmental media within the basin.

### 2.2. Sample Collection and Analysis

A total of 69 surface water samples, 79 soil samples, and 79 corresponding crop samples were collected in October 2024 (Figure S1). The soil sampling sites were primarily distributed in agricultural areas. The unrepresentative areas, such as ditches and garbage piles, were avoided. Each soil sample was a composite of five subsamples collected at depths of 0–20 cm. For the crop samples, edible portions from 5 to 15 representative plants per sampling site were collected and combined into one sample. Vegetables accounted for 96% of crop samples; grains and cotton accounted for 4%. Diseased and damaged plants were not sampled. The surface water samples were taken 0.3–0.5 m below the river surface near the soil sampling points.

Soil samples were air-dried, ground, and sieved through a 2 mm nylon mesh. The sieved soil was thoroughly mixed to homogeneity and placed in clean plastic bags for analysis. Crop samples were trimmed to remove visibly decayed outer leaves. The remaining crops were rinsed with deionized water, drained, and weighed. They were then oven-dried at 100–105 °C, milled, and stored for analysis. Soil and vegetable samples were digested by microwave digestion using nitric acid (65–68%), hydrofluoric acid (40%), and hydrochloric acid (37%). After digestion, Cr, Ni, Cu, Zn, Cd, Pb, As, Mo, V, and Sb were determined by inductively coupled plasma mass spectrometry (ICP-MS, Perkin-Elmer, Waltham, MA, USA). For water sampling, bottles were pre-rinsed 3–4 times with 10% nitric acid. After sampling, stabilizers were added and the bottles were sealed. All samples were stored at <4 °C until laboratory analysis. In water, Cr, Ni, Cu, Zn, Cd, Pb, As, Mo, and V were measured by ICP-MS (Perkin-Elmer, Waltham, MA, USA), and Sb was measured by hydride-generation atomic absorption spectrometry (HG-AAS, Perkin-Elmer, Waltham, MA, USA). To ensure accuracy and precision, 10% blank samples were included in each batch. Each site included two parallel samples, and each analysis was repeated three times. Recoveries for all elements were within the standard range of 90–110%.

### 2.3. Data Analysis

#### 2.3.1. Ecological Risk Assessment Indices

NI is a composite index used to assess the overall PTE contamination of soils. It reflects both the average condition and the most extreme element. NI is sensitive to the chosen geochemical background [37,38]. In this study, we evaluated PTEs across multiple media and, by analogy, applied NI to surface water and crops to characterize overall PTE contamination [36]. The single pollution index (PI) and NI are calculated as follows:

(1) $${PI}_{i}=\frac{C_{i}}{S_{i}},$$

(2) $$NI=\sqrt{\frac{{{PI}_{max}}^{2}+{{PI}_{average}}^{2}}{2}},$$

where PI*_i_* is the single pollution index for PTE *i*; NI is the comprehensive pollution index; and *C_i_* and *S_i_* are the measured concentration and the corresponding standard or background value for PTE *i*, respectively (Table S1). Based on the PI, the pollution levels are categorized as follows: safe (≤1), alert (1–2), slight (2–3), moderate (3–5), and severe (>5). The NI categories are as follows: safe (≤0.7), alert (0.7–1), slight (1–2), moderate (2–3), and severe (>3) [18].

The PERI was employed to assess the ecological risks posed by the PTEs in the surface water and soil, and the differences in the toxicity and environmental sensitivity of different elements were considered [39,40]. The PERI is calculated as follows:

(3) $$PERI=\sum {EI}_{i}=\sum \frac{C_{i}}{S_{i}}{\times T}_{r}^{i},$$

where EI*_i_* is the single ecological risk index for PTE *i*; PERI is the comprehensive ecological risk index; and *T_r_^i^* is the toxicity response coefficient for PTE *i* (Table S1). Based on the EI, the risk categories are as follows: low (<40), moderate (40–80), considerable (80–160), high (160–320), and extremely high (≥320). Based on the PERI, the risk categories are as follows: low (<150), moderate (150–300), considerable (300–600), high (600–1200), and extremely high (≥1200) [21,41].

#### 2.3.2. Health Risk Assessment Model

The HRA model recommended by the U.S. Environmental Protection Agency was employed to evaluate the health risks posed by the PTEs in the surface water and soils to adults and children [42]. PTEs primarily enter the human body through ingestion, dermal contact, and inhalation pathways. They accumulate over time and lead to chronic non-carcinogenic and/or carcinogenic health risks. The specific calculation equations are as follows:

(4) $${ADI}_{\mathrm{ing}}=\frac{C\times R_{ing}\times EF\times ED}{BW\times AT}\times 10^{-6},$$

(5) $${\mathrm{ADI}}_{\mathrm{der}}=\frac{C\times SA\times AF\times ABS\times EF\times ED}{BW\times AT}\times 10^{-6},$$

(6) $${ADI}_{\mathrm{inh}}=\frac{C\times R_{inh}\times EF\times ED}{PEF\times BW\times AT},$$

where *ADI_ing_*, *ADI_der_*, and *ADI_inh_* are the average daily intakes of PTEs via the ingestion, dermal contact, and inhalation pathways, respectively; *C* is the concentration of PTEs in different environmental media; *R_ing_* is the soil ingestion rate; *R_inh_* is the air inhalation rate; *EF* is the exposure frequency; *ED* is the exposure duration; *BW* is body weight; *AT* is the mean time; *SA* is the skin area; *AF* is the skin adherence factor; *ABS* is the dermal absorption factor; and *PEF* is the particle emission factor. The values of the parameters in these equations are presented in Table S2.

The non-carcinogenic health risk index (NRI) and carcinogenic health risk index (CRI) are calculated as follows:

(7) $${NRI}_{total}=\sum {NRI}_{i,j}=\sum \frac{{ADI}_{i,j}}{{RD}_{i,j}},$$

(8) $${CRI}_{total}=\sum {CRI}_{i,j}=\sum {ADI}_{i,j}\times {SF}_{i,j},$$

where NRI and CRI are the non-carcinogenic and carcinogenic health risk indices, respectively; *i* denotes the PTE; *j* denotes the exposure pathway; and *RD* and *SF* are the reference dose and slope factor, respectively (Table S3). NRI values above 1 indicate a possible non-carcinogenic risk, whereas values below 1 indicate an acceptable level. CRI values above 1 × 10^−4^ indicate that a carcinogenic risk to the population exists, while values below 1 × 10^−4^ indicate that heavy metals do not pose health risks to humans [43,44].

#### 2.3.3. Monte Carlo Simulation and Sensitivity Analysis

Monte Carlo simulation is a statistical method based on random sampling and probability distributions, and it can effectively characterize the uncertainties inherent in the HRA model [45,46]. Unlike traditional HRA models that use deterministic parameters, Monte Carlo simulation generates probabilistic distributions of predictive outcomes by repeatedly sampling the input parameters from predefined distributions, enabling quantitative evaluation of the uncertainty of the results [47]. Furthermore, this method facilitates sensitivity analysis to quantify the influences of individual input parameters on the prediction results [48,49]. In this study, Monte Carlo simulations were performed separately for the PTEs at every sampling site using the R software (v 3.5.0). A total of 10,000 iterations were conducted for each simulation, followed by sensitivity analyses based on the simulation outputs.

#### 2.3.4. Source Apportionment

PCA was employed to identify the relationships among the PTEs. PCA is a well-established method for pollution source apportionment. It requires fewer variables, which reduces the impact of multicollinearity on the results. PCA condenses complex environmental patterns into simpler processes and helps reveal the underlying environmental features. However, it has limitations. Its ability to handle nonlinear data is limited, and the dimensionality-reduction step can introduce distortion. Strong correlations between PTEs indicate a shared source or similar transport pathways, enabling assessment of the consistency of the PTE contamination sources across the study area [22,50]. In this study, PCA was also used to quantify the source-specific contributions of individual PTEs. Combined with the contributions of each PTE to the NRI and CRI calculated using the HRA model, the results clarify the contributions of different pollution sources to human health risks. The specific calculation equations are as follows:

(9) $${NQ}_{k}=\sum M_{i,k}\times {NP}_{i},$$

(10) $${CQ}_{k}=\sum M_{i,k}\times {CP}_{i},$$

where *NQ_k_* and *CQ_k_* are the contribution rates of pollution source *k* to the NRI and CRI, respectively; *M* is the contribution rate of PTE *i* to pollution source *k*; and *NP_i_* and *CP_i_* are the contribution rates of PTE *i* to the NRI and CRI, respectively.

## 3. Results

### 3.1. Characteristics of PTE Concentrations

Descriptive statistics of the PTE concentrations of the surface water, soil, and crops are presented in Table 1. In the surface water, the mean concentrations of Ni, Mo, and Sb all exceeded the threshold values stipulated in China’s Environmental Quality Standards for Surface Water. The coefficients of variation (CVs) of all 10 PTEs in the surface water exceeded 100%, indicating that their concentrations and spatial distributions were strongly influenced by anthropogenic activities. The CVs of Ni (690%), Cu (724%), and Zn (459%) were particularly high, suggesting that they had the most pronounced human impacts. In the soils, the mean concentrations of Mo and Sb were higher than the corresponding environmental background values. In particular, the mean concentration of Sb was approximately ten times that of the relevant background value. The CVs of Pb (37%), Cr (22%), Ni (23%), Cu (23%), Zn (20%), As (34%), Mo (49%), and V (21%) suggested that their distributions were jointly controlled by the geological background and anthropogenic inputs. Cd (63%) and Sb (84%) more clearly reflected the influence of human activities. Notably, the mean concentrations of Mo and Sb were above the relevant thresholds in both the surface water and soil. Thus, Cd and Sb warrant priority monitoring to prevent further accumulation. In the crops, the mean concentrations of Pb, Cr, and Ni were higher than the corresponding standard thresholds. The CVs of all of the PTEs exceeded 50%, indicating marked anthropogenic disturbance in the crops, and Mo (527%), Cd (121%), and Sb (105%) were the most prominent. Because there are no unified contamination assessment criteria for Mo and V in crops, in this study, we did not evaluate the crop contamination by these two elements.

### 3.2. Ecological Risk Analysis

#### 3.2.1. Pollution Analysis Based on the NI

**Figure 2:**
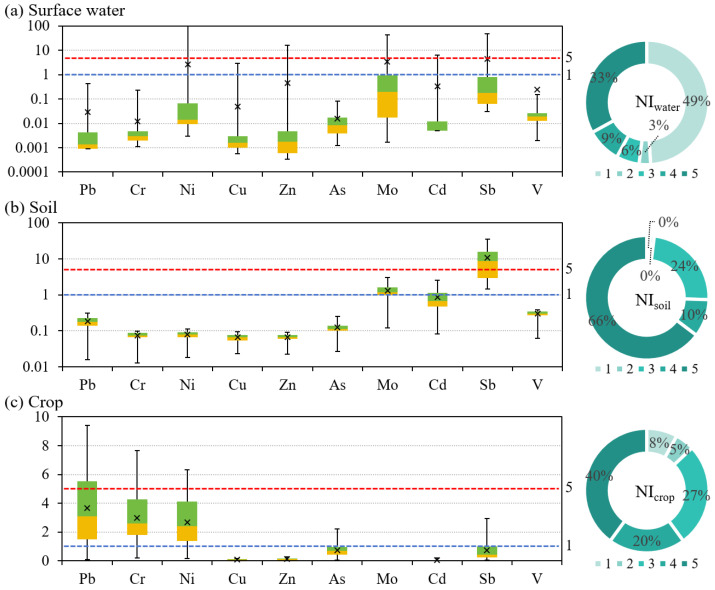
Statistics of the PI and NI values of different environmental media. The boxplots show the PI values. The blue dashed line indicates the alert threshold, and the red dashed line indicates the severe pollution threshold. The pie charts show the NI values, and pollution levels increase from light to dark.

The statistical results and spatial distributions of the NI and PI values of different environmental media are shown in Figure 2 and Figure S2. For the surface water, for a few samples, the Ni, Cu, Zn, Mo, Cd, and Sb values exceeded the alert threshold; however, the PI distributions of all of the PTEs were relatively concentrated below this threshold. Based on the NI results for the surface water, 33% of the samples fell within the severe pollution category. The high NI values were mainly distributed around the two mining areas. For the soils, Mo and Sb exhibited the most pronounced pollution, while the other PTEs were all within the safe range. The PI values of Sb were mainly above the severe pollution threshold, while those of Mo were mainly between the alert and severe pollution thresholds. According to the soil NI values, 66% of the samples were classified as severely polluted. The high NI values were primarily distributed in the upper and lower reaches of the study area. For the crops, Pb, Cr, and Ni were the principal pollutants, and the PI values were mostly clustered between the alert and severe pollution thresholds. The other elements were predominantly below the alert threshold. For the crops, 40% of the samples were classified as severely polluted, and the high NI values were concentrated in the upper and middle reaches of the study area. Mo and Sb were among the main pollutants in both the soil and surface water, while Pb, Cr, and Ni were the main pollutants in the crops. This phenomenon indicates that there were differences in the metal accumulation among the crops [51]. Moreover, the spatial distribution trends of the NI differed among the three environmental media. Although the surface water exhibited the widest range of NI values, the exceedances were confined to localized areas, resulting in a relatively low pollution level.

#### 3.2.2. Risk Analysis Based on PERI

**Figure 3:**
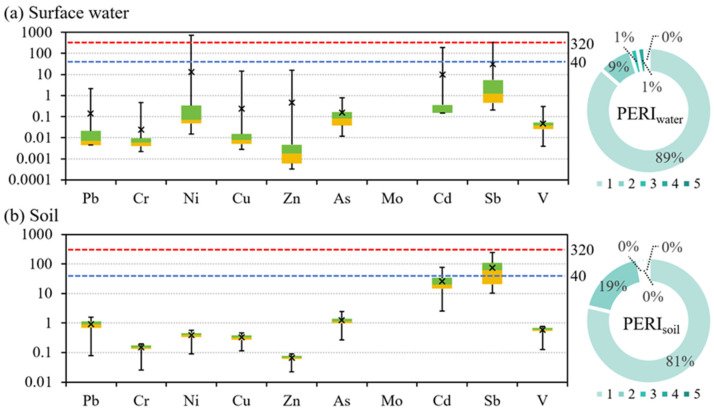
Statistics of the EI and PERI values of different environmental media. The boxplots show the EI values. The blue dashed line indicates the ecological-risk alert threshold, and the red dashed line indicates the extremely high ecological-risk threshold. The pie charts show the PERI values, and the risk levels increase from light to dark.

Figure 3 presents the statistical distributions of the PERI and EI values for the surface water and soil. For the surface water, the EI distributions of all of the PTEs were generally concentrated below the ecological-risk alert threshold. Relative to the PI results, the risk levels of Ni, Cu, Zn, Cd, and Sb all declined. For Ni, Cd, and Sb, only a few samples exhibited higher ecological risks, and the proportions exceeding the ecological-risk alert threshold were 3%, 7%, and 17%, respectively. For the surface water, the ecological risk was predominantly low (89%), and only a few samples had moderate (9%), considerable (1%), and high (1%) ecological risk levels. For the soil, the EI values for Sb were concentrated near the ecological-risk alert threshold, whereas those of the other PTEs were generally below this level. Based on the PI values, the risk levels of Cd and Sb declined; the proportions of the samples with PI values exceeding the ecological-risk alert threshold for Cd and Sb were 19% and 54%, respectively. Most of the soil samples (81%) had a low ecological risk, and the remainder of the samples (19%) had a moderate risk. According to Figure S2, the spatial pattern of the PERI of the surface water and soils was similar to that of the NI. The PERI results reflect both the influence of the measured concentrations of the samples and the weighting of different toxicity response coefficients in the PERI calculation.

### 3.3. Human Health Risk Analysis Based on Monte Carlo Simulation

**Figure 4:**
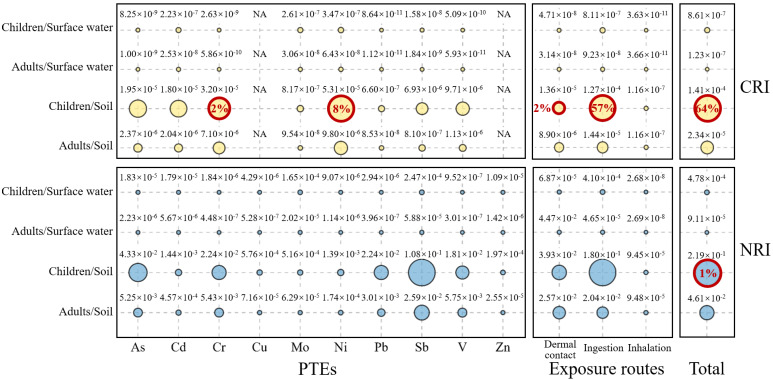
Mean NRI and CRI values for different environmental media. The red circles indicate unacceptable carcinogenic risk.

Using Monte Carlo simulation, 10,000 random simulations were conducted for each sampling point (surface water and soil) to determine the human health risk probability and mean values for different populations (adults and children). As shown in Figure 4, for both the surface water and soils, the mean NRI and CRI values of each PTE for both populations were below the corresponding risk thresholds. Among all of the PTEs, Sb had the highest NRI values, and Ni had the highest CRI values. Across the exposure pathways, for the surface water and soil, the ranking of the NRI and CRI for both populations was ingestion > dermal contact > inhalation. Compared with ingestion and dermal contact, inhalation via the oral-nasal route contributed negligibly and was unlikely to cause notable health risks. For the surface water, the mean NRI and CRI across the three pathways were all below their thresholds. In the soils, the mean NRI for both adults and children and the mean CRI for adults were also below the thresholds; however, for children, the mean CRI via ingestion was 1.27 × 10^−4^, indicating an unacceptable carcinogenic risk. Individual PTEs posed negligible to minor health risks. However, combined exposure to multiple PTEs elevated overall risk.

Although the surface water in the study area was relatively safe, the soil still had a certain probability of health risks. For the soils, children faced a certain probability of carcinogenic risk from Cr (2%) and Ni (8%). Based on the NI and PERI assessments, while Mo and Sb were the primary pollutants in the soils, according to Table S3 and Figure S3, Cr and Ni still posed health threats to children because of their higher concentrations and slope factors. In addition, for the soils, children faced 57% and 2% probabilities of carcinogenic risk via ingestion and dermal contact, respectively. Synthesizing across the PTEs and exposure pathways, the surface water presented neither non-carcinogenic nor carcinogenic risks for either population, and the soil presented neither non-carcinogenic nor carcinogenic risks for adults; however, children faced 1% and 64% probabilities of non-carcinogenic and carcinogenic risks, respectively. Figure S4 shows the probability distribution of the carcinogenic risk that the PTEs in the soils posed to children, which was similar to the spatial patterns of the NI and PERI.

**Figure 5:**
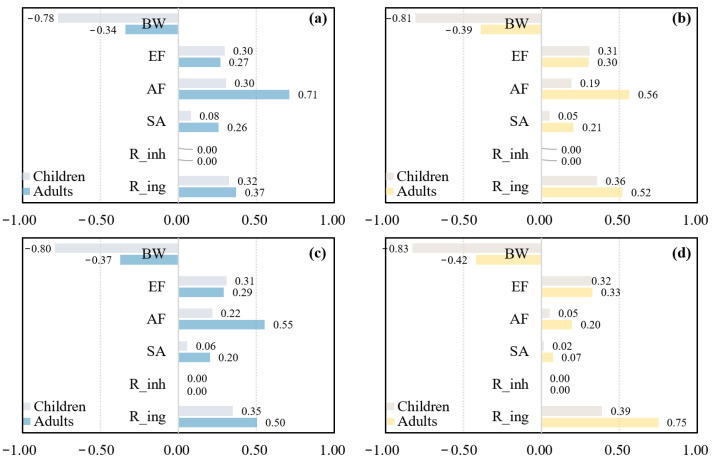
Sensitivity analysis of different parameters for adults and children: (**a**) NRI for both populations via soil; (**b**) CRI for both populations via soil; (**c**) NRI for both populations via surface water; and (**d**) CRI for both populations via surface water.

As shown in Figure 5, sensitivity analysis was conducted on the comprehensive health risks of the PTEs in soils and surface water. For adults, the parameter ranking was AF > R_ing_ > BW > EF > SA > R_inh_. For children, the ranking was BW > R_ing_ > EF > AF > SA > R_inh_. In particular, R_ing_, BW, and EF became more influential for adult CRI in surface water. For both NRI and CRI, BW showed negative sensitivity, indicating that appropriate weight gain could help reduce health risks to some extent. Compared with adults, the importance of BW increased and the importance of AF decreased for children, while SA and R_inh_ remained unchanged and had little effect. In summary, children faced higher non-carcinogenic and carcinogenic risks than adults, mainly due to their greater sensitivity to body weight and exposure time. Therefore, special attention should be paid to the protection of children, with a focus on the health risks posed by PTEs via the ingestion pathway.

In the vicinity of an iron-slag pile in Guangxi, China, the mean CRIs for children from soil, groundwater, and surface water were 6.79 × 10^−4^, 4.20 × 10^−6^, and 1.15 × 10^−6^, respectively [21]. The overall risk was high and mainly driven by slag stockpiles (PMF > 60%) [21]. In sulfide-mineralized soils, mean ingestion-pathway CRIs exceeded 1 × 10^−4^, indicating a serious potential risk [22]. Children had higher non-carcinogenic and carcinogenic risks than adults, consistent with our findings. In the downstream area of a stone-coal mine, children again faced higher risks than adults, with ingestion as the dominant exposure route. As and Cd posed greater carcinogenic risks to children, reaching 2.98% and 7.76%, respectively [43]. These results further support the conclusions of this study. Overall, the risk level in our study area is comparable to that of other typical mining districts in China. The key features are consistent: ingestion is the main pathway, and children are more susceptible.

### 3.4. Analysis of Pollution Sources Using PCA

**Figure 6:**
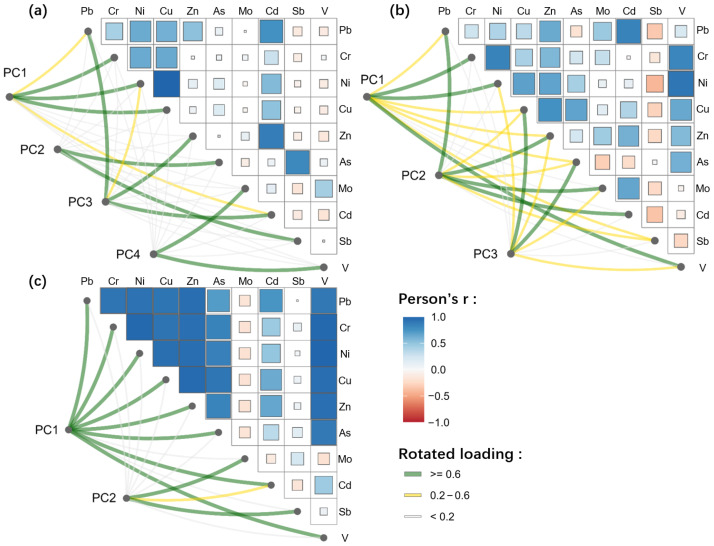
Source analysis of the PTEs based on correlation analysis and principal component analysis: (**a**) surface water, (**b**) soil, and (**c**) crops.

The study area hosted multiple types of mining enterprises, which were primarily concentrated within the two regions circled in red in Figure 1. Based on the Kaiser criterion (eigenvalue of >1), PCA was conducted separately for the PTEs in the surface water, soil, and crops (Figure 6). For the surface water, four principal components (PCs) were identified, and they explained a cumulative variance of 86%. PC1 was predominantly contributed by Cr, Ni, and Cu, and the high values were distributed in the upper reaches of the study area, coinciding with the concentrated mining zone. PC2 was mainly associated with As and Sb, and the high values were concentrated in the western mining area (I). This component was likely linked to antimony mining activities. PC3 was dominated by Pb, Zn, and Cd, and the high values were mainly distributed in the eastern mining area (II), suggesting a stronger influence from lead-zinc mining. PC4 was primarily contributed by Mo and V, and the high values were dispersed throughout the study area, indicating that its distribution was mainly controlled by the geological background rather than localized anthropogenic inputs. For the soils, three PCs were identified, accounting for a cumulative variance of 83%. PC1, dominated by Cr, Ni, and V, exhibited a dispersed distribution, primarily reflecting the influence of the geological background. PC2, contributed mainly by Pb, Zn, Mo, Cd, and Sb, exhibited a distribution that was strongly affected by mining activities. PC3 was primarily associated with Cu and As, and the high values were concentrated in the farmland adjacent to the main streams in the study area. Cu and As were major components of the pesticides and herbicides used, and these agricultural inputs accumulated in the soils through farming activities. The larger farmland areas and higher agricultural inputs around the main streams likely explain the presence of Cu and As [49,52]. For the crops, two PCs were identified, explaining a cumulative variance of 82%. PC1 was dominated by Pb, Cr, Ni, Cu, Zn, As, Cd, and V, and this was likely related to the fertilizers and pesticides used in the study area. PC2, contributed mainly by Mo and Sb, exhibited a distribution that was largely influenced by mining activities. Moreover, for the same PTEs, the absolute correlation coefficients across surface water, soil, and crops were below 0.22, indicating weak inter-media associations.

### 3.5. Priority Control Factors for Soil Pollution

**Figure 7:**
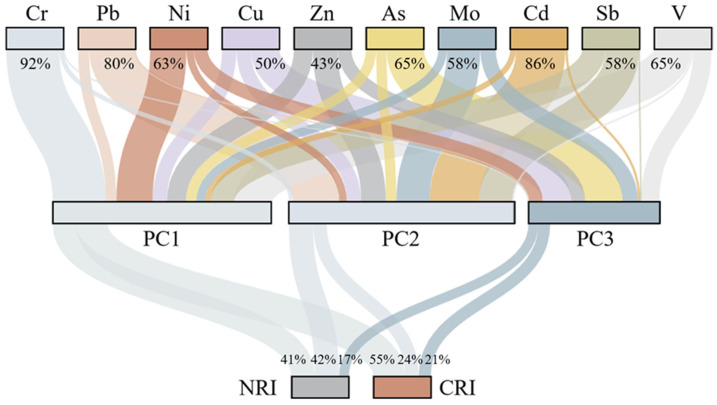
Relationship between PTEs, pollution sources, and health risks posed by soils to children.

According to the results of the HRA model, the health risks posed by the PTEs in the soil to children were markedly higher than the health risks posed by the PTEs to adults. Further analysis was conducted on the contributions of the different pollution sources of the soils to children’s health risks (Figure 7). For the non-carcinogenic risk, the contributions of the source-related principal components were ranked as follows: PC2 > PC1 > PC3. Natural (geogenic) sources accounted for 41%, mining-related sources accounted for 42%, and agricultural sources accounted for 17%. For the carcinogenic risk, the order of the contributions of the PCs was PC1 > PC2 > PC3. Natural (geogenic) sources accounted for 55%, mining-related sources accounted for 24%, and agricultural sources accounted for 21%. The geological background and mining pollution were important contributors to the soil-related health risks to children, underscoring the need for targeted prevention and control. Recommended measures include the rapid removal of mine wastes, co-processing in cement kilns, and complementary administrative measures such as preventing children from entering contaminated areas [21]. By quantifying the source-specific contributions to the health risks, in this study, we identified priority sources and actionable strategies for subsequent risk management.

## 4. Conclusions

In this study, we systematically evaluated the contamination levels, ecological risk, human health hazards, and potential sources of 10 PTEs (Zn, Pb, Cd, Cu, Ni, Mo, As, Cr, V, and Sb) across multiple environmental media (surface water, arable soils, and crops) in the Laoguan River Basin, China. The PTE concentrations differed markedly among the different media tested. Based on the NI and PERI, Mo and Sb were the principal pollutants in the soils and surface water, whereas Pb, Cr, and Ni were the dominant pollutants in the crops, with pronounced inter-crop variability of the metal accumulation. The HRA model revealed that the surface water generally posed a low risk, while the soil posed a substantial risk. The soils posed greater non-carcinogenic (1%) and carcinogenic (64%) risks to children than to adults, and ingestion was identified as the dominant exposure pathway, reflecting children’s higher sensitivity due to their lower body weight and longer exposure duration. The source apportionment results suggested that the surface water pollution was mainly linked to diverse mining activities; the soil pollution was jointly influenced by the geological background and mining and agricultural activities; and the crop contamination was primarily associated with mining and agricultural activities. Geological background and mining were identified as the major drivers of the elevated health risks for children. Overall, these findings underscore the urgent need for targeted risk management and site remediation, including combining engineering interventions with administrative controls, to safeguard local residents’ health and safety.

## Figures and Tables

**Table 1:** Descriptive statistics of the PTE concentrations of different environmental media.

Media	Item	Pb	Cr	Ni	Cu	Zn	As	Mo	Cd	Sb	V
Surface water (μg L^−1^)	Mean	2.86	1.22	51.52	48.34	918.12	1.57	235.05	3.21	21.91	1.20
	Max	42.10	23.50	2950.00	2910.00	31,300.00	7.99	3040.00	63.70	239.00	7.40
	Min	0.09	0.11	0.06	0.56	0.67	0.12	0.12	0.05	0.15	0.10
	SD ^1^	8.17	3.92	355.39	350.05	4214.48	1.83	610.58	11.35	51.66	1.34
	Skewness	3.50	5.03	8.21	8.27	6.08	1.99	3.30	3.95	2.75	3.55
	CV ^2^ (%)	286	320	690	724	459	117	260	353	236	112
Soils (mg kg^−1^)	Mean	21.79	14.87	7.85	6.53	16.58	3.70	0.73	0.26	9.62	22.87
	Max	37.42	19.85	11.41	9.27	22.60	7.43	1.75	0.76	31.89	30.06
	Min	1.91	2.58	1.78	2.30	5.67	0.80	0.07	0.03	1.32	4.83
	SD	7.99	3.34	1.82	1.50	3.37	1.26	0.36	0.16	8.08	4.70
	Skewness	−0.03	−1.12	−0.67	−0.48	−0.83	1.28	0.70	1.34	1.03	−1.44
	CV (%)	37	22	23	23	20	34	49	63	84	21
Crops (mg kg^−1^)	Mean	1.10	1.49	0.80	0.69	2.32	0.37	0.00	0.01	0.74	1.88
	Max	2.82	3.83	1.90	1.62	5.49	1.10	0.02	0.04	2.92	4.57
	Min	0.03	0.09	0.05	0.04	0.15	0.02	0.00	0.00	0.03	0.15
	SD	0.76	0.95	0.51	0.39	1.29	0.22	0.00	0.01	0.78	1.14
	Skewness	0.55	0.64	0.47	0.30	0.26	0.80	5.43	1.37	1.51	0.48
	CV (%)	69	64	63	57	56	61	527	121	105	61

^1^ Standard deviation; ^2^ Coefficient of variation.

## Data Availability

The raw data supporting the conclusions of this article will be made available by the authors on request.

## Supplementary Materials

The following supporting information can be downloaded at: https://www.mdpi.com/article/10.3390/toxics13110952/s1, Table S1: Details of the parameters for the pollution assessment indexes; Table S2: Details of the parameters for the health risk assessment model; Table S3: Values of the reference dose and slope factor for PTEs; Figure S1: Locations of the sampling sites: a) surface water sampling points; and b) soil and crop sampling points; Figure S2: Spatial distributions of pollution levels in different environmental media based on the inverse distance weighting method; Figure S3: Box plots of PTE concentrations in different environmental media; Figure S4: CRI evaluation results for children in soils. (a) Probability distribution of Ni; (b) probability distribution through ingestion; (c) probability distribution of the total CRI; (References [21,22,41,43,53,54,55,56,57,58,59,60,61,62,63,64] are cited in the Supplementary Materials).
