# Supplementary material for: Source-Oriented Health Risk Assessment of Potentially Toxic Elements in the Water-Soil-Crop System Using Monte Carlo Simulation: A Case Study of the Laoguan River Basin, China
Source: Toxics. 2025 Nov 4;13(11):952. doi: 10.3390/toxics13110952 (PMC12655975; doi:10.3390/toxics13110952)
Supplement: Supplementary file 1 [file toxics-13-00952-s001.zip › toxics-3928222-supplementary.pdf]

Source-oriented health risk assessment of potentially toxic elements in the water-soil-crop system using Monte Carlo simulation: A case study of the Laoguan River Basin, China

List

Table S1. Details of the parameters for the pollution assessment indexes.

Table S2. Details of the parameters for the health risk assessment model.

Table S3. Values of the reference dose ($mg\cdot kg^{-1}\cdot day^{-1}$) and slope factor ($kg\cdot day\ mg^{-1}$) for PTEs.

Figure S1. Locations of the sampling sites: a) surface water sampling points; and b) soil and crop sampling points.

Figure S2. Spatial distributions of pollution levels in different environmental media based on the inverse distance weighting method.

Figure S3. Box plots of PTE concentrations in different environmental media.

Figure S4. CRI evaluation results for children in soils. a) Probability distribution of Ni; b) probability distribution through ingestion; c) probability distribution of the total CRI.

Table S1. Details of the parameters for the pollution assessment indexes.

Parameter		Pb	Cr	Ni	Cu	Zn	As	Mo	Cd	Sb	V
S [a]	Water (mg L^{-1}) [b]	0.1	0.1	0.02	1	2	0.1	0.07	0.01	0.005	0.05
	Soil (mg kg^{-1}) [c]	120	200	100	100	250	30	0.57	0.3	0.9	77.5
	Crop (mg kg^{-1}) [d]	0.3	0.5	0.3	10	20	0.5	-	0.2	1	-
T_r [e]		5	2	5	5	1	10	-	30	7	2

Notes: [a] The standard or background value. [b] Values are taken from MEE (2002). [c] Mo, Sb and V values are taken from Xi et al. (2021), and values for the other PTEs are taken from MEE (2018). [d] Pb, Cr, As and Cd values are taken from NHC (2022); Ni and Sb values are taken from Nie et al. (2016) and Zhang et al. (2022), respectively; Cu and Zn values are taken from NHC (1994) and NHC (1991) respectively. [e] The toxicity response coefficient, of which values are taken from Hakanson (1980), Xu et al. (2008), and Wang et al. (2018).

Table S2. Details of the parameters for the health risk assessment model.

Symbol	Parameter	Unit	Adult	Child
R_{ing}	Soil ingestion rate	mg day^{-1}	T [a] (4, 30, 52)	T (66, 103, 161)
R_{inh}	Air inhalation rate	m^3 day^{-1}	LN [b] (16.57, 4.05)	LN (7.19, 1.62)
EF	Exposure frequency	day $year^{-1}$	T (180, 350, 365)	T (180, 350, 365)
ED	Exposure duration	year	24	6
BW	Body weight	kg	N [c] (61.9, 11.31)	T (5.25, 29.3, 56.8)
AT	Mean Time	day	8760	2190
SA	Skin area	cm^2	T (760, 1530, 3820)	T (430, 860, 2160)
AF	Skin adherence factor	mg cm^{-2}	LN (0.49, 0.54)	LN (0.58, 1.2)
ABS	Dermal absorption factor	Unitless	0.001	0.001
PEF	Particle emission factor	m^3 kg^{-1}	1.36×10^9	1.36×10^9

Notes: [a] Triangular distribution; [b] Lognormal distribution; [c] Normal distribution. Values for the parameters are taken from Dai et al. (2024) and Hu et al. (2024).

Table S3. Values of the reference dose ($mg \cdot kg^{-1} \cdot day^{-1}$) and slope factor ($kg \cdot day$ mg^{-1}) for PTEs.

Metal	RD_{ing}	RD_{der}	RD_{inh}	SF_{ing}	SF_{der}	SF_{inh}
Pb	3.50E-03	5.25E-04	3.52E-03	8.50E-03	4.20E-02	8.50E-03
Cr	3.00E-03	6.00E-05	2.86E-05	5.00E-01	2.00E+01	4.20E+01
Ni	2.00E-02	5.40E-03	2.06E-02	1.70E+00	4.25E+01	8.40E-01
Cu	4.00E-02	1.20E-02	4.00E-02	-	-	-
Zn	3.00E-01	6.00E-02	3.00E-01	-	-	-
As	3.00E-04	1.23E-04	3.00E-04	1.50E+00	3.66E+00	1.51E+01
Mo	5.00E-03	1.90E-03	5.00E-03	3.20E-01	3.20E-01	3.20E-01
Cd	1.00E-03	1.00E-05	1.00E-03	2.00E+01	3.80E-01	6.30E+00
Sb	4.00E-04	8.00E-06	4.00E-04	2.07E-01	2.07E-01	2.07E-01
V	7.00E-03	7.00E-05	7.00E-03	1.22E-01	1.22E-01	1.22E-01

Notes: Values for the parameters are taken from Ferreira and Miguel (2005), Ma et al. (2020), Dai et al. (2024), and Zhang et al. (2024).

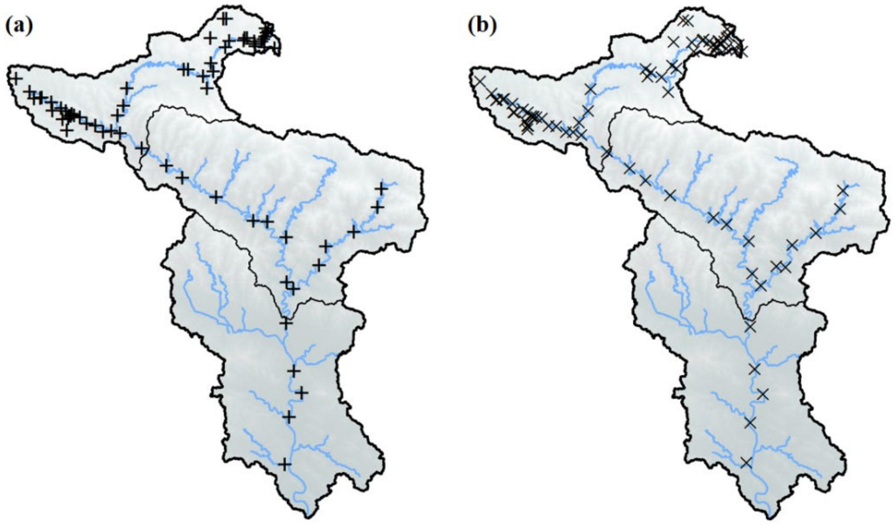

Figure S1. Locations of the sampling sites: a) surface water sampling points; and b) soil and crop sampling points.

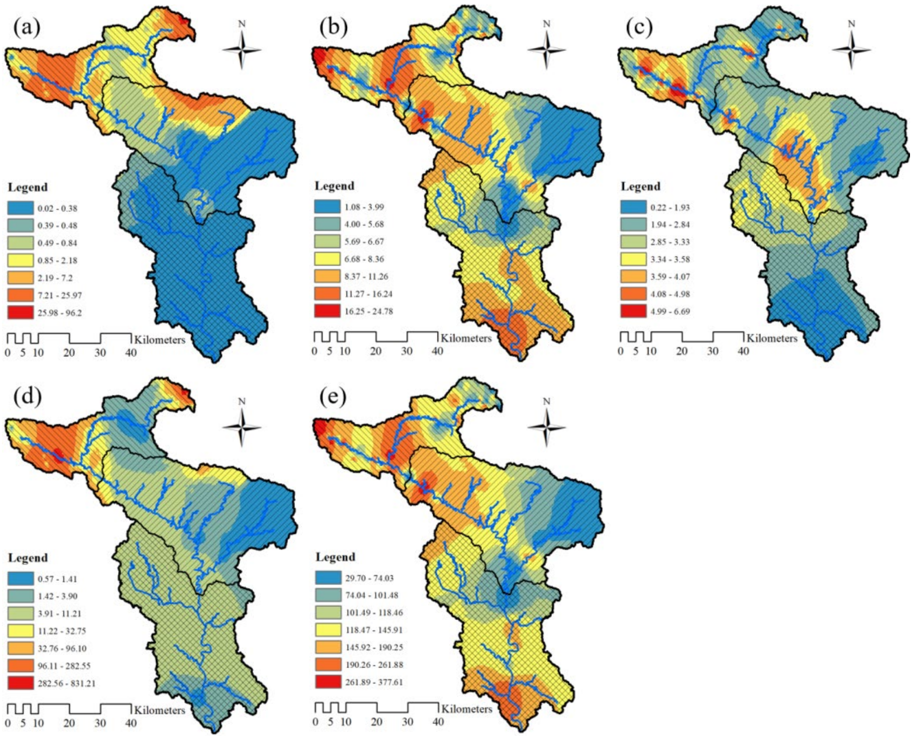

Figure S2. Spatial distributions of pollution levels in different environmental media based on the inverse distance weighting method: a) NI of surface water; b) NI of soils; c) NI of crops; d) PERI of surface water; and e) PERI of soils.

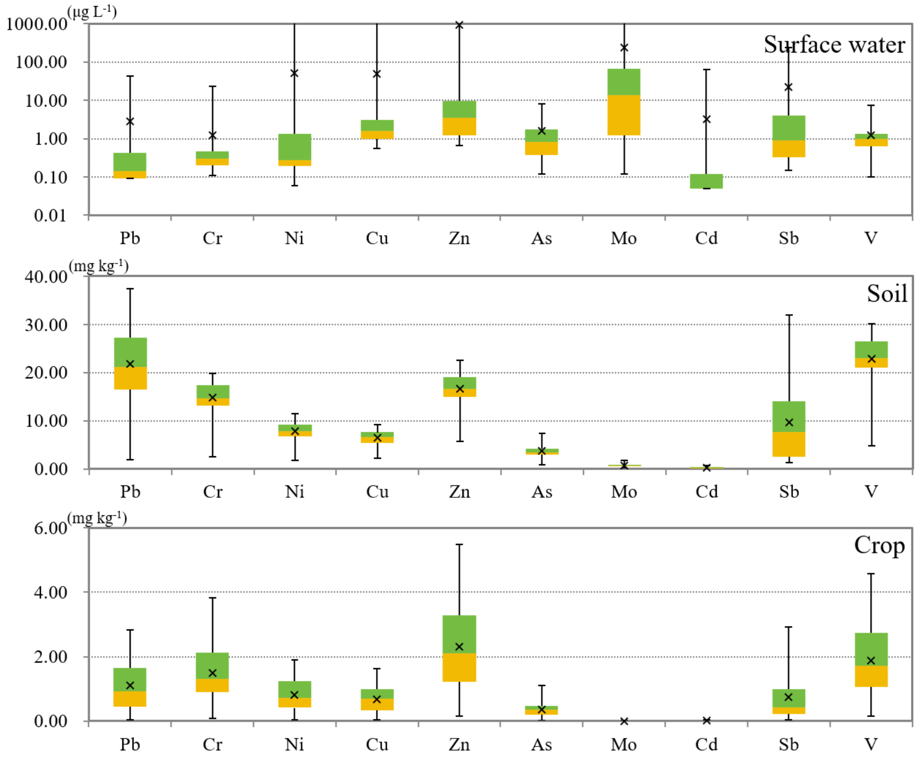

Figure S3. Box plots of PTE concentrations in different environmental media.

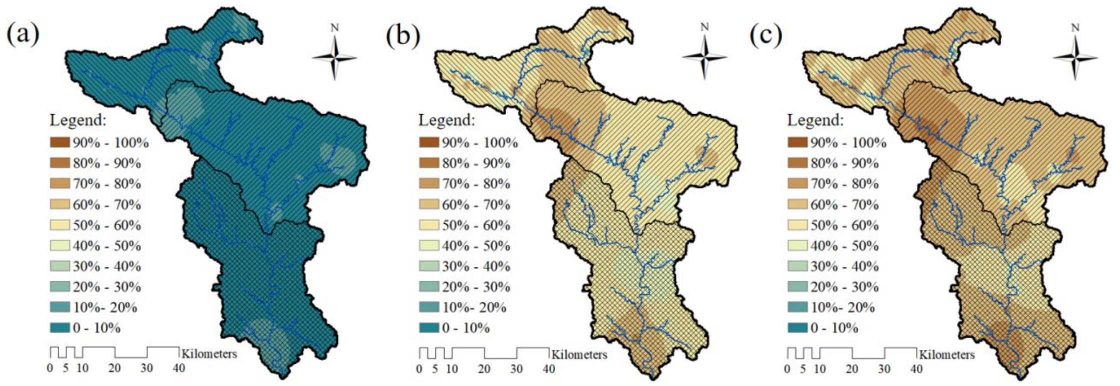

Figure S4. CRI evaluation results for children in soils. a) Probability distribution of Ni; b) probability distribution through ingestion; c) probability distribution of the total CRI.

Reference:

Dai, X.; Liang, J.; Shi, H.; et al. Health risk assessment of heavy metals based on source analysis and Monte Carlo in the downstream basin of the zishui. *Environ. Res.* **2024**, *245*, 117975.

Ferreira, B.L.; Miguel, E.D. Geochemistry and risk assessment of street dust in Luanda, Angola: A tropical urban environment. *Atmos. Environ.* **2005**, *39*, 4501-4512.

Hakanson, L. An ecological risk index for aquatic pollution control: a sedimentological approach. *Water Res.* **1980**, *14*, 975-1001.

Hu, H.; Zheng, H.; Liu, F.; et al. Heavy metal contamination assessment and source attribution in the Vicinity of an iron slag pile in Hechi, China: Integrating multi-medium analysis. *Environ. Res.* **2024**, *263*, 120206.

Ma, L.; Xiao, T.; Ning, Z.; et al. Pollution and health risk assessment of toxic metal(loid)s in soils under different land use in sulphide mineralized areas. *Sci. Total Environ.* **2020**, *724*, 138176.

MEE (Ministry of Ecology and Environment). Soil environmental quality risk control standard for soil contamination of agricultural land. GB 15618-2018, **2018**.

MEE (Ministry of Ecology and Environment). Environmental quality standards for surface water. GB 3838-2002, **2002**.

NHC (National Health Commission). Tolerance limit of copper in foods. GB 15199-94, **1994**.

NHC (National Health Commission). Tolerance limit of zine in foods. GB 16106-91, **1991**.

NHC (National Health Commission). National standard for food safety–Limits of contaminants in foods. GB 2762-2022, **2022**.

Nie, X.L.; Wang, M.J.; Liu, Y.; et al. Surveillance on the contents of nickel in commercial vegetables and health risk evaluation in Shaanxi province. *Chin. J. Health Lab. Tec.* **2016**, *26*, 1065-1068.

Wang, N.N.; Wang, A.H.; Kong, L.H.; et al. Calculation and application of Sb toxicity coefficient for potential ecological risk assessment. *Sci. Total Environ.* **2018**, *610-611*, 167-174.

Xi, X.H.; Hou, Q.Y.; Yang, Z.F.; et al. Big data based studies of the variation features of Chinese soil's background value versus reference value: A paper written on the occasion of Soil Geochemical Parameters of China's publication. *Geophys. Geochem. Explor.* **2021**, *45*, 1095-1108.

Xu, Z.Q.; Ni, S.J.; Tuo, X.G.; et al. Calculation of heavy metals' toxicity coefficient in the evaluation of potential ecological risk index (in Chinese). *Environ. Sci. Technol.* **2008**, *31*, 112-115.

Zhang, K.; Chang, S.; Tu, X.; et al. Heavy metals in centralized drinking water sources of the Yangtze River: A comprehensive study from a basin-wide perspective. *J. Hazard. Mater.* **2024**, *469*, 133983.

Zhang, L.; Song, B.; Huang, F.Y.; et al. Characteristics of Antimony migration and transformation and pollution evaluation in a soil-crop system around a Tin mine in Hunan Province. *Environ. Sci.* **2022**, *43*, 1558-1566.
